# STOUT: SMILES to IUPAC names using neural machine translation

**DOI:** 10.1186/s13321-021-00512-4

**Published:** 2021-04-27

**Authors:** Kohulan Rajan, Achim Zielesny, Christoph Steinbeck

**Affiliations:** 1 Institute for Inorganic and Analytical Chemistry, Friedrich-Schiller-University Jena, Lessingstr. 8, 07743 Jena, Germany; 2 Institute for Bioinformatics and Chemoinformatics, Westphalian University of Applied Sciences, August-Schmidt-Ring 10, 45665 Recklinghausen, Germany

**Keywords:** Neural machine translation, Chemical language, IUPAC names, SMILES, DeepSMILES, SELFIES, Deep neural network, Attention mechanism, Recurrent neural network

## Abstract

Chemical compounds can be identified through a graphical depiction, a suitable string representation, or a chemical name. A universally accepted naming scheme for chemistry was established by the International Union of Pure and Applied Chemistry (IUPAC) based on a set of rules. Due to the complexity of this ruleset a correct chemical name assignment remains challenging for human beings and there are only a few rule-based cheminformatics toolkits available that support this task in an automated manner. Here we present STOUT (**S**MILES-**TO**-I**U**PAC-name **t**ranslator), a deep-learning neural machine translation approach to generate the IUPAC name for a given molecule from its SMILES string as well as the reverse translation, i.e. predicting the SMILES string from the IUPAC name. In both cases, the system is able to predict with an average BLEU score of about 90% and a Tanimoto similarity index of more than 0.9. Also incorrect predictions show a remarkable similarity between true and predicted compounds.

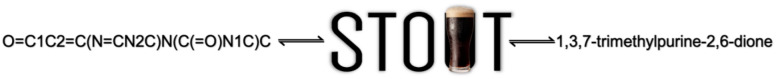

## Introduction

Assigning names to chemical compounds so that an author can refer to them in the text of a scientific article, book or patent has a long history. In the early days and even still today, such names were often chosen based on physicochemical or perceptible properties, but also named after species, people, named after fictional characters, related to sex, bodily functions, death and decay, religion or legend, or other [1]. Usually, this makes it impossible to conclude from the name to the chemical structure of the compound. To overcome this dilemma, the International Union of Pure and Applied Chemistry (IUPAC) established a set of rules and guidelines for chemical nomenclature [2–5] so that a systematic name can be generated from the structure and substructures of a chemical compound and vice versa. Often, more than one systematic IUPAC name can be generated for the same compound: Therefore, the IUPAC introduced the IUPAC preferred name in their current edition of the Blue Book, preferring one of the possible names over all others.

Other types of string representations of molecules, such as SMILES [6], InChI [7], SYBYL line notation [8], Wiswesser line notation [9], and SMARTS [10] are more concise forms of line representations. While in principle being human-readable, these representations are primarily designed to be understood by machines. Thus, they are not commonly used in text to denominate chemical compounds for recognition by human readers, but have been incorporated into many major open-source and proprietary cheminformatics toolkits.

IUPAC name generation, due to its algorithmic complexity and the large set of rules, is missing in many cheminformatics toolkits in general. For a human, IUPAC name generation for more than a handful of molecules is cumbersome. People, therefore, resort to the few available automatic tools for IUPAC name generation.

Among the available and reliable solutions are the “molconvert” software, a command-line program in Marvin Suite 20.15 from ChemAxon (https://www.chemaxon.com) [11]. It is available for researchers under an academic license. Open-source programs such as the Chemistry Development Kit (CDK) [12], RDKit [13], or Open Babel [14] do not (yet) provide any algorithms that can automate the process of IUPAC naming for molecules.

With this work, we report a proof-of-concept application of Neural Machine Translation (NMT) for the conversion of machine-readable chemical line notations into IUPAC names and vice versa. A large training set was generated with ChemAxon’s molconvert software and we would like to emphasise that this work would not have been possible without the generous offer by ChemAxon for the academic scientific community to use their software for free. We also like to point out that the purpose of this work is not to make ChemAxon’s tool obsolete. As a deterministic tool, it will continue to be the first choice for practical naming tasks in databases.

For the work presented here, we were inspired by Google’s multiple NMT models and came up with the idea to build a **S**MILES-**TO**-I**U**PAC-name **t**ranslator called STOUT. STOUT was developed based on language translation and language understanding. We treated the two chemical representations as two different languages—each SMILES string and corresponding IUPAC name was treated as two different sentences that have the same meaning in reality.

All these language models can only achieve greater than 90% accuracy with sufficient data to train them on. The majority of state-of-the-art language translation models are trained on millions of words and sentences to achieve such high levels of accuracy. Moreover, to train such large models in an adequate amount of time dedicated and powerful machine learning hardware is required. In this work, we report substantially shortened training times for our models using Google’s Tensor Processing Units (TPU).

## Methods

Using deep machine learning methods such as NMT for SMILES-to-IUPAC-name translation is a completely data-driven task so that high-quality data from a reliable source is mandatory. In this work, datasets were created for SMILES-to-IUPAC-name translation as well as for IUPAC-name-to-SMILES translation respectively.

### Data

All molecules were obtained from PubChem [15], one of the openly available large small molecule databases, where the entire PubChem database was downloaded from its FTP site in SDF format. Using the CDK, explicit hydrogens were removed from the molecules and their topological structures were converted to canonical SMILES strings. The obtained 111 million molecules were filtered according to the ruleset of our previous DECIMER work [16], i.e. molecules must

- have a molecular weight of fewer than 1500 Da,
- not possess any counter ions,
- contain only C, H, O, N, P, S, F, Cl, Br, I, Se and B,
- not contain any hydrogen isotopes (D, T),
- have between 3 and 40 bonds,
- not contain any charged group,
- contain implicit hydrogens only, except in functional groups,

to arrive at a dataset of 81 million molecules. These selected SMILES strings were converted into IUPAC names using Chemaxon’s molconvert software, a command-line program in Marvin Suite 20.15 from ChemAxon (https://www.chemaxon.com).

**Fig.1:**
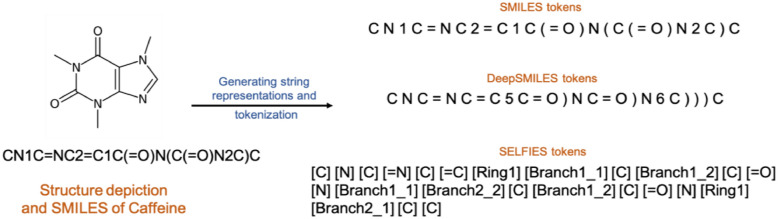
SMILES, DeepSMILES and SELFIES split into tokens which are separated by a space character

Using SMILES strings directly for training Neural Networks (NN) may cause various problems due to their intricate structure which is difficult to split into separate meaningful tokens necessary for the machine input. To tackle this problem, two other representations are available, DeepSMILES [17] and SELFIES [18]. For a discussion of the problems of string tokenization for deep learning, we refer our readers to those two publications. Our results confirm the superiority of SELFIES for the task discussed here and in our work on Optical Chemical Entity Recognition [16]. Thus, for this work all SMILES strings were converted into SELFIES using a custom python script (Fig. 1).

Two datasets were constructed, a 30 million and 60 million molecule set with SELFIES and corresponding IUPAC names, where the 60 million sets contained all 30 million molecule entries of the former. Every SELFIES string and IUPAC name was split into separate tokens using the space character as a delimiter. SELFIES were split according to a closed square bracket “]” and an open square bracket “[”. For IUPAC names a small set of rules was applied to split them uniformly: After every,

- open bracket “(”, “{” and “[”,
- close bracket “)”, “}” and “]”,
- dash symbol “-”,
- full stop “.”,
- comma “,”

and after every word in the following list,

- mono,di,tri,tetra,penta,hexa,hepta,octa,nona
- deca,oxo,methyl,hydroxy,benzene,oxy,chloro,cyclo,amino,bromo,hydro,fluoro
- methane,cyano,amido,ethene,phospho,amide,butane,carbono,hydro,sulfane,butane,sulfino
- iodo,ethane,ethyne,bi,imino,nitro,butan,idene,sulfo,carbon,propane,ethen,acetaldehyde,benzo,oxa,nitroso,hydra,iso

a space character was added as a delimiter. After adding the delimiter, the dataset was padded to fit the maximum length of 48 characters for SELFIES strings and 78 characters for IUPAC name strings, a “start” token was added to each string to indicate its beginning, and an “end” token was added at the end of the string. The strings were tokenized and saved into small TFRecord files for training with GPUs or TPUs. Finally, two SELFIES-to-IUPAC-name datasets and two IUPAC-name-to-SELFIES datasets—with 30 million (exactly 30,000,128) and 60 million (exactly 60,000,256) molecules each - were generated.

### Network

The NMT network follows the implementation reported by Google for their language translation models, which itself is built on the network designed by Luong et al. [19] for neural machine translation, using a soft attention mechanism developed by Bahdanau et al. [20]. It is based on an autoencoder–decoder architecture and is written on Python 3 with Tensorflow 2.3.0 [21] at the backend. The encoder network and the decoder network use Recurrent Neural Networks (RNNs) with Gated Recurrent Units (GRU). The input strings are passed to the encoder and the output strings to the decoder. The encoder network generates the encoder output and the encoder hidden state. The attention weight is calculated by the attention mechanism implemented in the network. Encoder output with attention weights then creates the context vector. Meanwhile, the decoder output is passed through an embedding layer. The output generated by the embedding layer and the context vector is concatenated and passed on to the GRUs of the decoder. An Adam optimizer with a learning rate of 0.0005 is applied and sparse categorical cross-entropy is used to calculate the loss with a modified loss function. A batch size of 256 Strings is used for a GPU and a global batch size of 1024 Strings for a TPU where the global batch size is divided between the nodes.

**Fig. 2:**
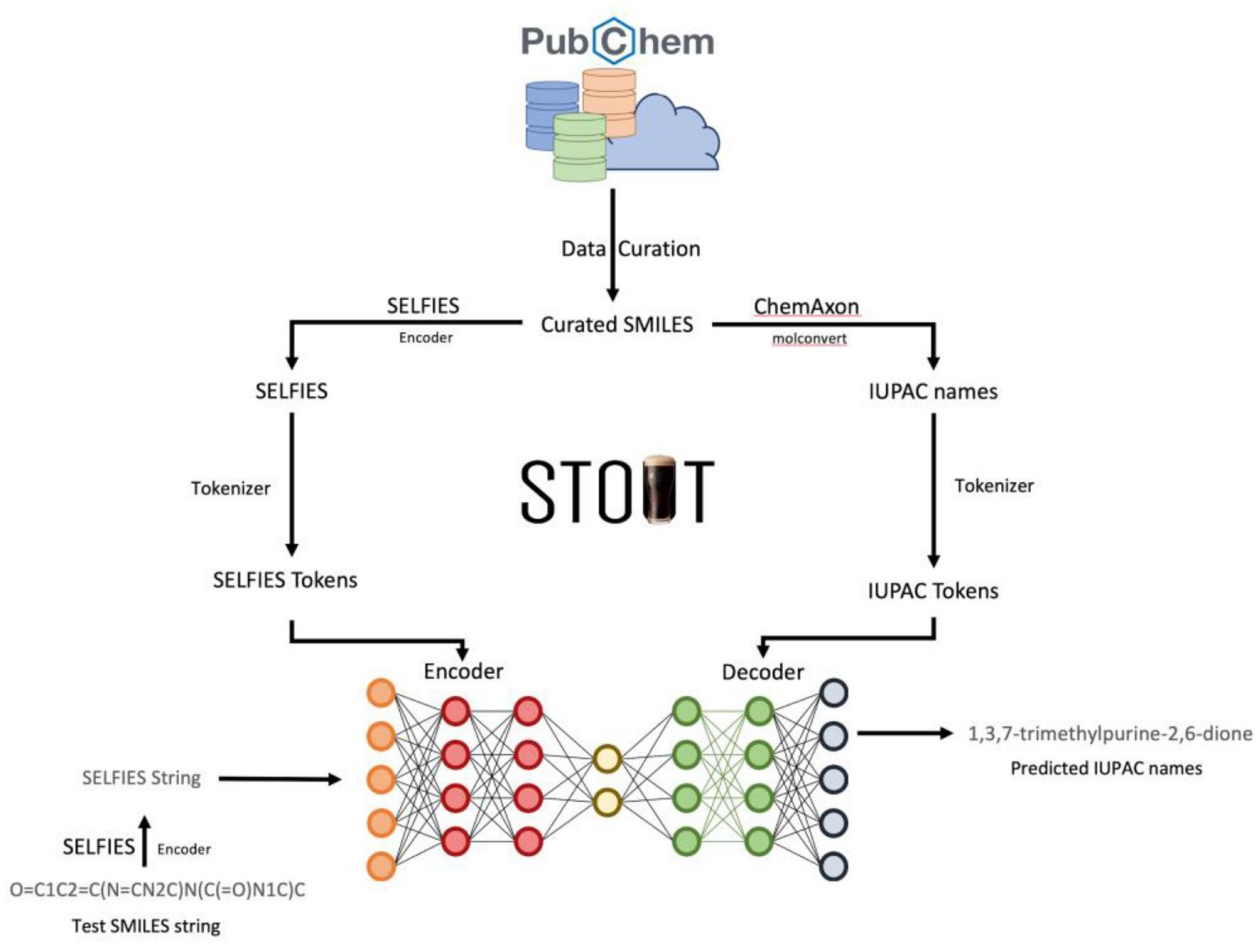
STOUT architecture for SMILES-to-IUPAC-name translation

For SELFIES-to-IUPAC-name and IUPAC-name-to-SELFIES translation the same network architecture is used with the input/output datasets simply being swapped. Figure 2 shows the STOUT architecture for SMILES-to-IUPAC-name translation.

### Model training

For large datasets, training a neural network efficiently is a challenging task. As an initial test, the network was trained with 15 million molecules on a server with an nVidia Tesla V100 GPU, 384GB of RAM, and two Intel(R) Xeon(R) Gold 6230 processors. The average training epoch was evaluated to be about 27 h so that training of larger datasets appeared to be prohibitive. With more than 100 epochs of training time used in our training described below, those 27 h per epoch translate into almost 4 months of training time, with multiples of that for training with 30 million or 60 million structures. Thus, the training scripts were modified to use Tensor Processing Units (TPUs) available on the Google cloud using the Tensorflow distributed training API. A corresponding training with TPU V3-8 units (with 8 nodes each) reduced the average training epoch to about 2 h.

### Model testing

To evaluate the models' performance, a test set of 2.2 Million molecules was used, which was not present in the 30 million and the 60 million molecules training sets. A uniform and highly similar frequency distribution of unique SELFIES tokens in training and test data were ensured by corresponding test molecule selection. The SELFIES-to-IUPAC-name translation and the reverse IUPAC-name-to-SELFIES translation were tested with the same set.

To assess the predictive accuracy BLEU scoring [22] was used (see Appendix for details). Also, Tanimoto similarities were calculated between original and predicted strings using PubChem fingerprints. For the predictions of IUPAC names as an output, the IUPAC names were re-converted to SMILES using OPSIN 2.5 [23] and canonicalised using the CDK, with the resulting SMILES being utilized for Tanimoto similarity calculations.

## Results and discussion

### Computational considerations

Table 1 shows the number of unique SELFIES/IUPAC-name tokens for both data sets. Note that the 30 million and the larger 60 million molecules datasets have the same number of tokens. To keep the same number of tokens we removed the least occurring tokens from both sets using a cutoff. In contrast, the SELFIES token set size is smaller than that of the IUPAC name tokens because the IUPAC names cover a far greater language space.

**Table 1:** Number of unique SELFIES and IUPAC-name tokens for each dataset

Dataset size	Number of SELFIES tokens	Number of IUPAC tokens
30 Million	27	1190
60 Million	27	1190

**Fig. 3:**
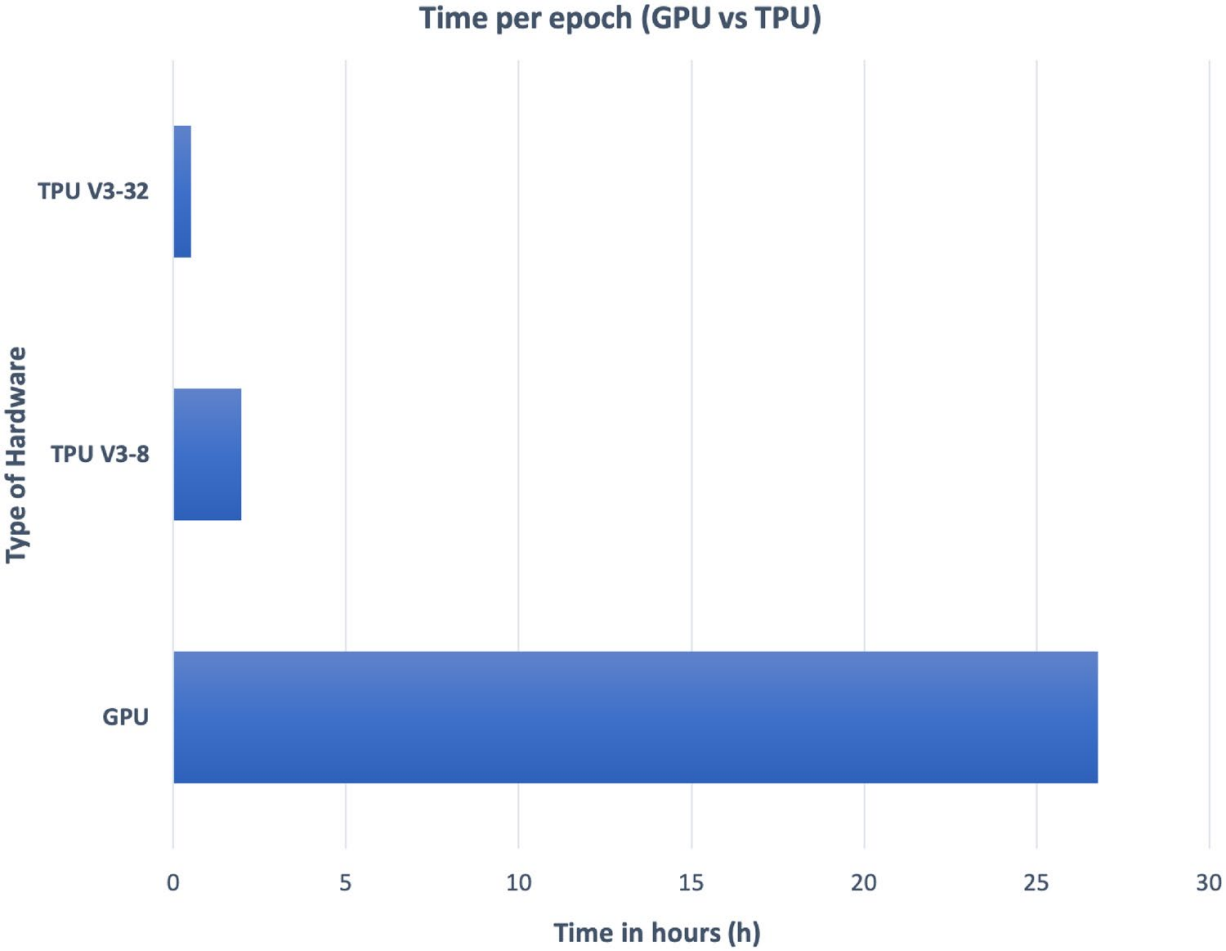
Average training time per epoch on different hardware (lower is better)

We used a 15 Mio training dataset to compare the training speed between a GPU and TPUs. Training 15 Million molecules on a TPU V3-8 requires 2 h per epoch which is 13 times faster than using a GPU V100. Using a TPU V3-32 allows for an additional 4 times faster performance in comparison to a TPU V3-8 and is 54 times faster compared to a GPU V100, see Fig. 3.

**Fig. 4:**
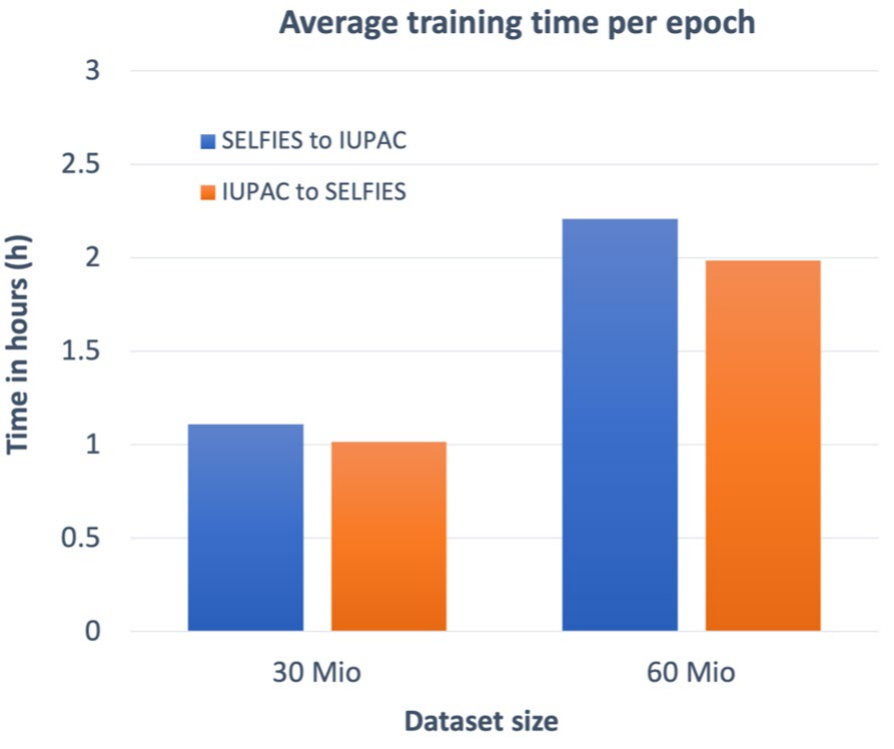
Average training time per epoch for different datasets using TPU V3-8

Figure 4 shows the different training times per epoch of the different datasets on TPU V3-8 units where all models were trained for more than 100 epochs until convergence. The difference between the SELFIES-to-IUPAC-name and IUPAC-name-to-SELFIES training is caused by the different number of I/O tokens of each dataset: For the SELFIES-to-IUPAC-name translation, the output tokens are derived from the IUPAC names whereas for the IUPAC-name-to-SELFIES translation the output tokens are taken from SELFIES strings. Since SELFIES strings are smaller and less complex than IUPAC name strings the IUPAC-name-to-SELFIES translation is faster.

### Test results

#### SELFIES-to-IUPAC-name translation

Table 2 summarizes the average and individual BLEU scores for the 30 million and the 60 million molecules dataset. A predicted string with a BLEU score of 1.0 means a score of 1.0 using the NLTK sentence BLEU scoring function[24] and they are mostly identical strings (see Appendix).

**Table 2:** BLEU scores analysis

Training dataset size	30 Mio	60 Mio
Average BLEU score	0.89	0.94
Total number of strings with BLEU 1.0	52.48%	66.65%
BLEU-1	0.92	0.95
BLEU-2	0.90	0.94
BLEU-3	0.88	0.93
BLEU-4	0.86	0.92

Compared to the 30 million molecules dataset, a model trained with 60 million molecules makes better predictions, as demonstrated by all BLEU score types.

To assess the network’s ability to learn “chemistry” we calculated the Tanimoto similarities between the predicted and the original molecules by translating the original and the predicted IUPAC names back to SMILES strings using OPSIN and canonicalised the retranslated SMILES using the CDK. We used the CDK with Pubchem fingerprints to calculate the Tanimoto similarity indices. The IUPAC names that OPSIN was able to translate back to SMILES strings were counted as valid IUPAC names while the others were counted as invalid. Only the valid IUPAC-name-to-SMILES translations were used for the Tanimoto similarity calculations. The average Tanimoto similarity was measured on valid IUPAC-name-to-SMILES translations. Additionally, both Tanimoto similarity calculations were readjusted to the number of data points present on the test dataset (see Table 3). We also computed full isomorphism matches using InChIs and found that 98% of all Tanimoto similarity 1.0 cases were full graph isomorphisms.

**Table 3:** Tanimoto similarities

Training dataset size	30 Mio	60 Mio
Invalid IUPAC names	21.41%	14.50%
Valid IUPAC names	78.59%	85.50%
Tanimoto 1.0 count on the total test dataset	58.36%	72.33%
Tanimoto 1.0 count on valid IUPAC names	74.26%	84.59%
Average Tanimoto (measured for total test dataset)	0.75	0.83
Average Tanimoto (measured for valid IUPAC names)	0.96	0.98

The invalid IUPAC names are the ones that were rejected by OPSIN and could not be converted into SMILES. This inability is the result of errors of the IUPAC names being predicted. In most cases, the IUPAC-name-to-SMILES translation failed because

- they did not contain a comma,
- some of them were missing a close bracket symbol corresponding to the open bracket symbol,
- the valence of an atom was wrong,
- a certain block of text was uninterpretable,
- they failed to assign all bonds correctly,
- of a disagreement between lengths of bridges and alkyl chain length
- of long names with repeating words.

Table 4 presents a few examples of IUPAC names that could not be converted to SMILES strings with an explanation of the failure.

**Table 4:** Failed IUPAC-name-to-SMILES translations

IUPAC names	Reason for failure (OPSIN error messages)
1. *N*-[6-(2,3-diaminopropylidene)-1-methyl-1,2,4a,5,6,8a-hexahydroquinolin-6-yl]-*N*-methylpropanamide	Atoms are in an unphysical valency state. Element: C valency: 5
2. 2-[({[(3-ethoxypropyl)amino]({[2-(2-fluorophenyl)ethyl]amino})methylidene}amino)-*N*,*N*-dimethylacetamide	Unmatched opening bracket found
3. 3'-(propan-2-yl)-2',3',4',5',6',7',8',8'a-octahydro-2'H-spiro[imidazole-4,1'-indolizin]-2-amine	The following being uninterpretable: 2',3',4',5',6',7',8',8'
4. ({2',6'-difluoro-2',6'-dimethyl-[1,1'-biphenyl]-4-yl}methyl)(propyl)amine	Failed to assign all double bonds
5. 1,4,5-trimethyl-1-[1,2-dimethylpropyl)-2-methyl-1-propylbicyclo[12.2.1]tetradeca-1,5-diene	Disagreement between lengths of bridges and alkyl chain length

**Fig. 5:**
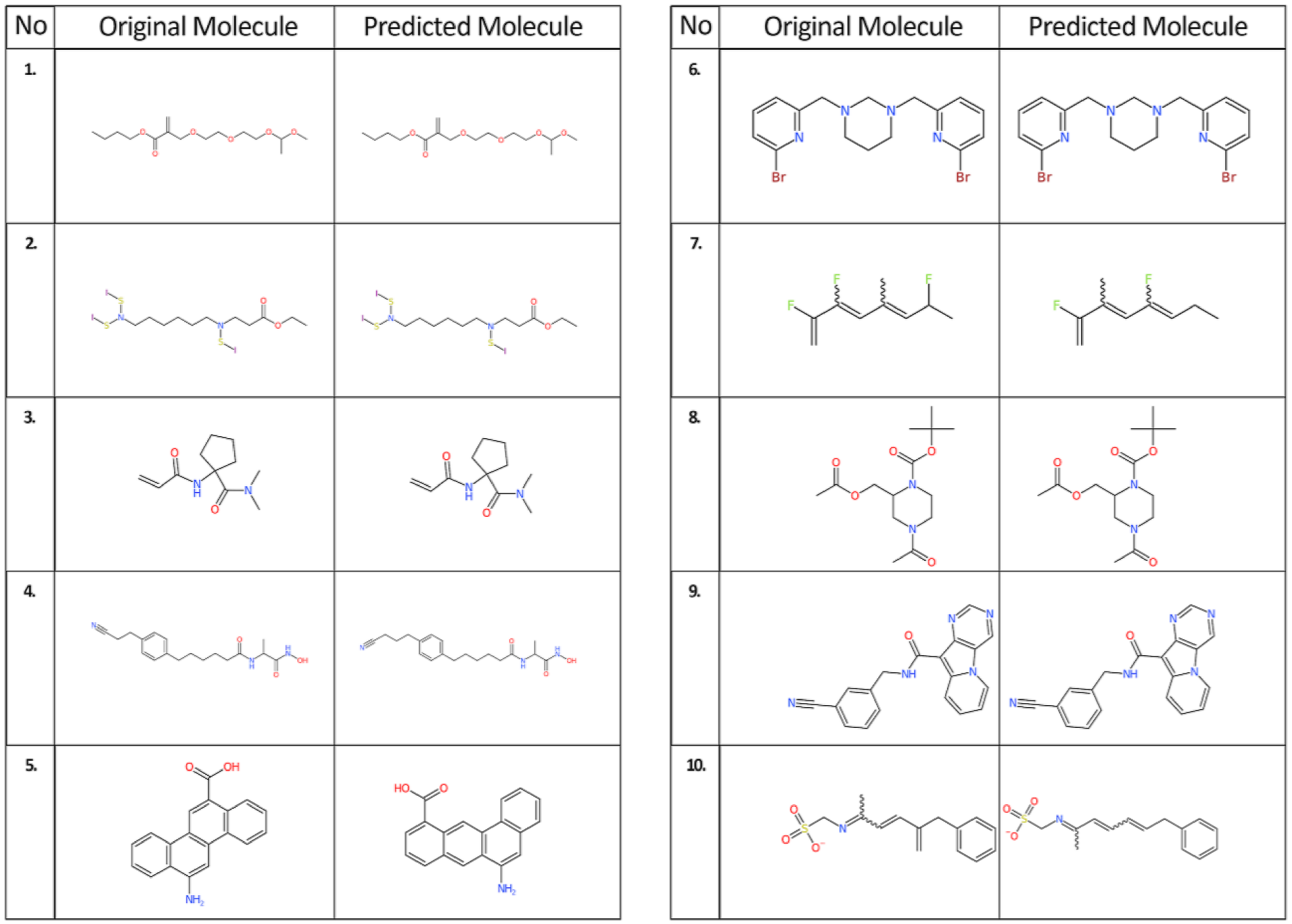
Chemical structures depicted with the CDK depiction generator for predictions with Tanimoto similarity 1.0 but low BLEU score

**Fig. 6:**
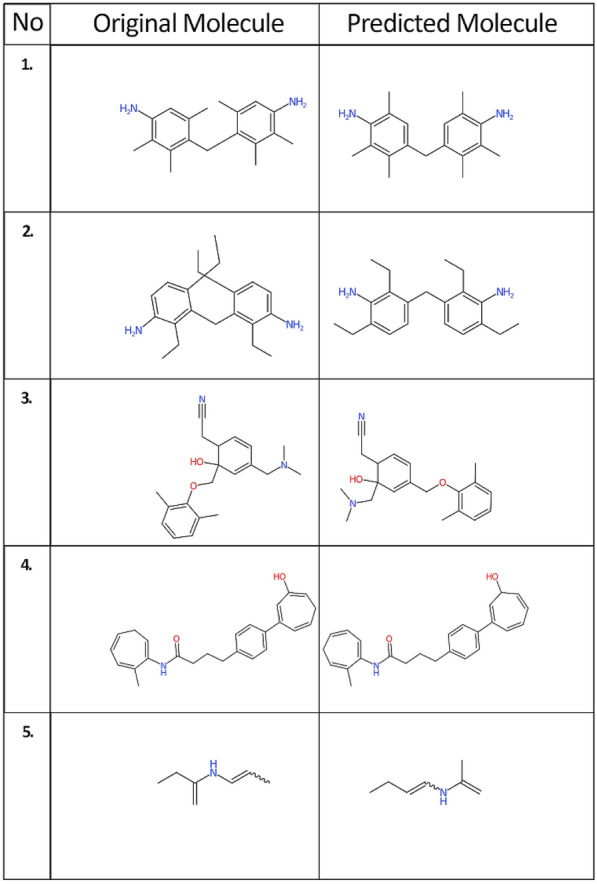
Chemical structures depicted with the CDK depiction generator for predictions with BLEU score 1.0 but Tanimoto similarity less than 1.0

The Tanimoto similarity index 1.0 count with 72% (60 million molecules set) of the test data is already remarkable but the average Tanimoto similarity of 0.83 (60 million molecules set) suggests that an “understanding” of the “language of chemistry” emerged. Also, it becomes obvious that the number of predictions with a Tanimoto similarity of 1.0 is greater than the number of predictions with a BLEU score of 1.0, see Table 5: Although there are different IUPAC names, using OPSIN to re-translate these names led to SMILES representations with similar or even identical chemical graphs, see Figure 5. This also illustrates the extent to which the model is capable to successfully generalise the information of the training data. We found that only five predictions had a Tanimoto similarity index less than 1.0 but a BLEU score of 1.0, see Table 6 and Fig. 6.

**Table 5:** Predicted IUPAC name strings with a Tanimoto similarity index of 1.0 but a low BLEU score

No.	IUPAC names		BLEU Score	IUPAC names translated into SMILES using OPSIN		Tanimoto similarity Index
	Original	Predicted		Original	Predicted	
1	butyl3-methyl-12-methylidene-2,4,7,10-tetraoxatridecan-13-oate	butyl2-({2-[2-(1-methoxyethoxy)ethoxy]ethoxy}methyl)prop-2-enoate	0.00	O=C(OCCCC)C(=C)COCCOCCOC(OC)C	O=C(OCCCC)C(=C)COCCOCCOC(OC)C	1.0
2	ethyl3-[1,10-diiodo-9-(iodosulfanyl)-1,10-dithia-2,9-diazadecan-2-yl]propanoate	ethyl3-({6-[bis(iodosulfanyl)amino]hexyl}(iodosulfanyl)amino}propanoate	0.10	O=C(OCC)CCN(SI)CCCCCCN(SI)SI	O=C(OCC)CCN(SI)CCCCCCN(SI)SI	1.0
3	*N*,*N*dimethyl-1-(prop-2-enamido)cyclopentane-1-carboxamide	*N*-[1-(dimethylcarbamoyl)cyclopentyl]prop-2-enamide	0.24	O=C(C=C)NC1(C(=O)N(C)C)CCCC1	O=C(C=C)NC1(C(=O)N(C)C)CCCC1	1.0
4	6-[4-(2-cyanoethyl)phenyl]-*N*-[1-(hydroxycarbamoyl)ethyl]hexanamide	2-{6-[4-(3-cyanopropyl)phenyl]hexanamido}-*N*-hydroxypropanamide	0.32	N#CCCC1=CC=C(C=C1)CCCCCC(=O)NC(C(=O)NO)C	N#CCCCC1=CC=C(C=C1)CCCCCC(=O)NC(C(=O)NO)C	1.0
5	12-aminochrysene-6-carboxylicacid	6-aminotetraphene-11-carboxylicacid	0.41	O=C(O)C1=CC=2C=3C=CC=CC3C(N)=CC2C=4C=CC=CC41	O=C(O)C1=CC=CC2=CC=3C(N)=CC=4C=CC=CC4C3C=C21	1.0
6	1,3-bis[(6-bromopyridin-2-yl)methyl]-1,3-diazinane	2-bromo-6-({3-[(6-bromopyridin-2-yl)methyl]-1,3-diazinan-1-yl}methyl)pyridine	0.50	BrC=1N=C(C=CC1)CN2CN(CC3=NC(Br)=CC=C3)CCC2	BrC=1N=C(C=CC1)CN2CN(CC3=NC(Br)=CC=C3)CCC2	1.0
7	2,3,7-trifluoro-5-methylocta-1,3,5-triene	2,5-difluoro-3-methylocta-1,3,5-triene	0.61	FC(=C)C(F)=CC(=CC(F)C)C	FC(=C)C(=CC(F)=CCC)C	1.0
8	tert-butyl4-acetyl-2-[(acetyloxy)methyl]piperazine-1-carboxylate	tert-butyl2-[(acetyloxy)methyl]-4-acetylpiperazine-1-carboxylate	0.72	O=C(OC(C)(C)C)N1CCN(C(=O)C)CC1COC(=O)C	O=C(OC(C)(C)C)N1CCN(C(=O)C)CC1COC(=O)C	1.0
9	*N*-[(3-cyanophenyl)methyl]pyrimido[4,5-b]indolizine-10-carboxamide	*N*-[(3-cyanophenyl)methyl]-5H-pyrimido[4,5-b]indolizine-10-carboxamide	0.83	N#CC1=CC=CC(=C1)CNC(=O)C=2C=3N=CN=CC3N4C=CC=CC24	N#CC1=CC=CC(=C1)CNC(=O)C=2C=3N=CN=CC3N4C=CC=CC24	1.0
10	(5-benzylhexa-3,5-dien-2-ylidene)aminomethanesulfonate	(6-benzylhexa-3,5-dien-2-ylidene)aminomethanesulfonate	0.92	O=S(=O)([O-])CN=C(C=CC(=C)CC=1C=CC=CC1)C	O=S(=O)([O-])CN=C(C=CC=CCC=1C=CC=CC1)C	1.0

**Table 6:** Predicted IUPAC name strings with a BLEU score of 1.0 but a low Tanimoto similarity index

No.	IUPAC names		BLEU Score	IUPAC names translated into SMILES using OPSIN		Tanimoto similarity Index
	Original	Predicted		Original	Predicted	
1	4-[(4-amino-2,3,6-trimethylphenyl)methyl]-2,3,5-trimethylaniline	4-[(4-amino-2,3,5-trimethylphenyl)methyl]-2,3,6-trimethylaniline	1.0	NC=1C=C(C(=C(C1C)C)CC=2C(=CC(N)=C(C2C)C)C)C	NC1=C(C=C(C(=C1C)C)CC2=CC(=C(N)C(=C2C)C)C)C	0.97
2	3-[(3-amino-2,6-diethylphenyl)methyl]-2,4-diethylaniline	3-[(3-amino-2,4-diethylphenyl)methyl]-2,6-diethylaniline	1.0	NC1=CC=C(C(=C1CC)CC=2C(=CC=C(N)C2CC)CC)CC	NC=1C(=CC=C(C1CC)CC2=CC=C(C(N)=C2CC)CC)CC	0.92
3	2-{4-[(dimethylamino)methyl]-6-[(2,6-dimethylphenoxy)methyl]-6-hydroxycyclohexa-2,4-dien-1-yl}acetonitrile	2-{4-[(2,6-dimethylphenoxy)methyl]-6-[(dimethylamino)methyl]-6-hydroxycyclohexa-2,4-dien-1-yl}acetonitrile	1.0	N#CCC1C=CC(=CC1(O)COC=2C(=CC=CC2C)C)CN(C)C	N#CCC1C=CC(=CC1(O)CN(C)C)COC=2C(=CC=CC2C)C	0.93
4	4-[4-(3-hydroxycyclohepta-1,3,6-trien-1-yl)phenyl]-*N*-(7-methylcyclohepta-1,4,6-trien-1-yl)butanamide	4-[4-(3-hydroxycyclohepta-1,4,6-trien-1-yl)phenyl]-*N*-(7-methylcyclohepta-1,3,6-trien-1-yl)butanamide	1.0	O=C(NC1=CCC=CC=C1C)CCCC=2C=CC(=CC2)C=3C=CCC=C(O)C3	O=C(NC1=CC=CCC=C1C)CCCC=2C=CC(=CC2)C=3C=CC=CC(O)C3	0.95
5	(but-1-en-2-yl)(prop-1-en-1-yl)amine	(but-1-en-1-yl)(prop-1-en-2-yl)amine	1.0	C=C(NC=CC)CC	C=C(NC=CCC)C	0.97

#### IUPAC-name-to-SELFIES translation

The IUPAC-name-to-SELFIES translation was tested with the same 2.2 million test molecules as the SELFIES-to-IUPAC-name model before, but in reverse order. To use OPSIN as a performance measure, we analyzed our test set using OPSIN. It was able to convert 98.31% of IUPAC names generated by the molconvert algorithm back to SMILES and 96.24% were found to show a Tanimoto 1.0 similarity, see Table 7 for details. Table 8 summarizes the average BLEU score, the calculated BLEU scores, and the Tanimoto similarities that were carried out on the test molecules for IUPAC-name-to-SELFIES translation.

**Table 7:** Analysis on test set using OPSIN

OPSIN analysis on test set	Values
Invalid IUPAC names	1.69%
Valid IUPAC names	98.31%
Tanimoto 1.0 count on the total test dataset	97.89%
Tanimoto 1.0 count on valid IUPAC names	96.24%
Average Tanimoto (measured for total test dataset)	0.99
Average Tanimoto (measured for valid IUPAC names)	0.98

**Table 8:** Average BLEU scores, BLEU Scores, and Tanimoto similarity calculations

	30 Mio	60 Mio
Average BLEU score	0.90	0.94
Total number of predicted strings with BLEU 1.0	46.78%	68.47%
BLEU-1	0.94	0.97
BLEU-2	0.91	0.95
BLEU-3	0.89	0.94
BLEU-4	0.85	0.92
Tanimoto calculations		
Average Tanimoto similarity index	0.89	0.94
Number of predicted strings with Tanimoto 1.0	52.27%	73.26%

**Fig. 7:**
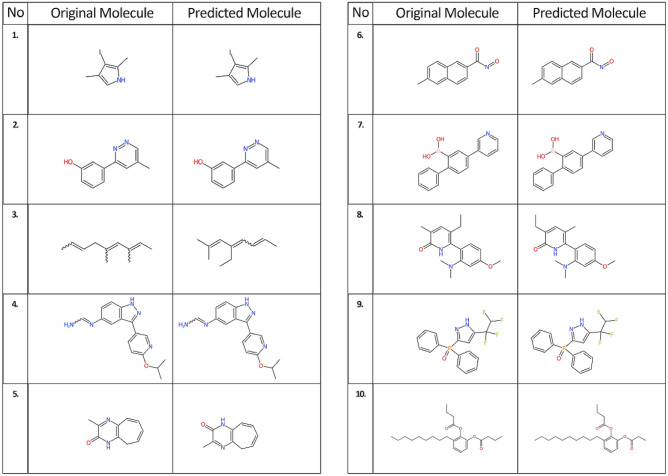
Chemical structures depicted with the CDK depiction generator for predictions with Tanimoto similarity 1.0 and low BLEU score

The larger 60 million molecules dataset again performs better than the 30 million molecules dataset. Invalid SELFIES do not occur because all the predicted SELFIES were retranslated into SMILES without any error. Again, the predictions with Tanimoto similarity index 1.0 exceed those with BLEU scores 1.0. The reason for this is that BLEU is calculated by mapping word to word for an original and predicted SELFIES string while Tanimoto similarity is calculated according to the corresponding chemical structure, see Table 9 and Figure 7. To improve these results, more molecules with the same set of unique tokens would be needed. We also saw that 860 out of 2.2 million molecules (0.0003%) had BLEU 1.0 but a slightly lower Tanimoto similarity index because of slight differences in the chemical structures.

**Table 9:** Predicted SELFIES with low BLEU scores and Tanimoto similarity 1.0

No.	SELFIES		BLEU Score	SELFIES decoded back into SMILES		Tanimoto similarity Index
	Original	Predicted		Original	Predicted	
1.	[I][C][C][Branch1_2][Branch1_3][=C][N][C][Expl=Ring1][Branch1_1][C][C]	[I][C][=C][Branch1_1][Branch1_3][N][C][=C][Ring1][Branch1_1][C][C]:	0.00	IC=1C(=CNC1C)C	IC=1C(=CNC1C)C	1.0
2.	[O][C][C][=C][C][=C][Branch1_1][Ring2][C][Expl=Ring1][Branch1_2][C][N][=N][C][=C][Branch1_1][Ring2][C][Expl=Ring1][Branch1_2][C]	[O][C][=C][C][=C][C][Branch1_2][Ring2][=C][Ring1][Branch1_2][C][=N][N][=C][C][Branch1_2][Ring2][=C][Ring1][Branch1_2][C]:	0.18	OC=1C=CC=C(C1)C=2N=NC=C(C2)C	OC=1C=CC=C(C1)C=2N=NC=C(C2)C	1.0
3.	[C][Branch1_2][=C][=C][C][C][Branch1_2][Branch2_1][=C][C][Branch1_2][Ring1][=C][C][C][C][C]	[C][Branch1_1][=N][C][=C][Branch1_1][Branch1_3][C][=C][Branch1_1][C][C][C][C][C][=C][C]:	0.21	C(=CCC(=CC(=CC)C)C)C	C(C=C(C=C(C)C)CC)=CC	1.0
4.	[N][=C][C][Branch1_2][N][=C][C][=C][Ring1][Branch1_2][O][C][Branch1_1][C][C][C][C][=N][N][C][C][=C][C][Branch1_1][Ring2][N][=C][N][=C][C][Ring1][N][Expl=Ring1][Branch2_2]	[N][Branch1_2][Ring1][=C][N][C][C][=C][C][N][N][=C][Branch1_1][P][C][C][=N][C][Branch1_1][Branch1_3][O][C][Branch1_1][C][C][C][=C][C][Expl=Ring1][Branch2_3][C][Expl=Ring1][#C][C][Expl=Ring2][Ring1][Ring1]:	0.32	N1=CC(=CC=C1OC(C)C)C2=NNC=3C=CC(N=CN)=CC23	N1=CC(=CC=C1OC(C)C)C2=NNC=3C=CC(N=CN)=CC23	1.0
5.	[O][=C][N][C][=C][Branch1_1][Branch1_2][N][=C][Ring1][Branch1_2][C][C][=C][C][=C][C][Ring1][Branch2_3]	[O][=C][N][C][C][=C][C][=C][C][C][Expl=Ring1][Branch1_3][N][=C][Ring1][O][C]:	0.45	O=C1NC2=C(N=C1C)C=CC=CC2	O=C1NC=2C=CC=CCC2N=C1C	1.0
6.	[O][=N][C][Branch1_2][C][=O][C][C][=C][C][=C][C][Branch1_2][Branch2_2][=C][C][=C][Ring1][Branch1_2][C][Expl=Ring1][Branch2_3][C]	[O][=N][C][Branch1_2][C][=O][C][=C][C][=C][C][=C][Branch1_1][Branch2_2][C][=C][C][Ring1][Branch1_2][=C][Ring1][Branch2_3][C]:	0.53	O=NC(=O)C=1C=CC2=CC(=CC=C2C1)C	O=NC(=O)C=1C=CC2=CC(=CC=C2C1)C	1.0
7.	[O][B][Branch1_1][C][O][C][=C][C][Branch1_2][=C][=C][C][=C][Ring1][Branch1_2][C][=C][C][=C][C][=C][Ring1][Branch1_2][C][=C][N][=C][C][=C][Ring1][Branch1_2]	[O][B][Branch1_1][C][O][C][C][=C][Branch1_1][=C][C][=C][C][Expl=Ring1][Branch1_2][C][C][=C][C][=C][C][Expl=Ring1][Branch1_2][C][=C][N][=C][C][=C][Ring1][Branch1_2]:	0.60	OB(O)C1=CC(=CC=C1C2=CC=CC=C2)C3=CN=CC=C3	OB(O)C1=CC(=CC=C1C2=CC=CC=C2)C3=CN=CC=C3	1.0
8.	[O][=C][N][C][Branch2_1][Ring1][C][C][C][=C][C][Branch1_1][Ring1][O][C][=C][C][Expl=Ring1][Branch2_1][N][Branch1_1][C][C][C][=C][Branch1_1][Branch1_2][C][=C][Ring1][P][C][C][C]	[O][=C][N][C][Branch2_1][Ring1][C][C][=C][C][=C][Branch1_1][Ring1][O][C][C][=C][Ring1][Branch2_1][N][Branch1_1][C][C][C][=C][Branch1_1][Branch1_3][C][=C][Ring1][P][C][C][C]:	0.71	O=C1NC(C=2C=CC(OC)=CC2N(C)C)=C(C=C1C)CC	O=C1NC(C=2C=CC(OC)=CC2N(C)C)=C(C=C1CC)C	1.0
9.	[O][=P][Branch2_1][Ring1][Branch1_2][C][=N][N][C][Branch1_2][Ring2][=C][Ring1][Branch1_1][C][Branch1_1][C][F][Branch1_1][C][F][C][Branch1_1][C][F][F][Branch1_1][Branch2_2][C][C][=C][C][=C][C][Expl=Ring1][Branch1_2][C][C][=C][C][=C][C][Expl=Ring1][Branch1_2]	[O][=P][Branch1_1][Branch2_2][C][C][=C][C][=C][C][Expl=Ring1][Branch1_2][Branch1_1][Branch2_2][C][C][=C][C][=C][C][Expl=Ring1][Branch1_2][C][=N][N][C][Branch1_2][Ring2][=C][Ring1][Branch1_1][C][Branch1_1][C][F][Branch1_1][C][F][C][Branch1_1][C][F][F]:	0.86	O=P(C1=NNC(=C1)C(F)(F)C(F)F)(C=2C=CC=CC2)C=3C=CC=CC3	O=P(C1=NNC(=C1)C(F)(F)C(F)F)(C=2C=CC=CC2)C=3C=CC=CC3	1.0
10.	[O][=C][Branch2_1][Ring1][=N][O][C][=C][C][=C][C][Branch1_2][N][=C][Ring1][Branch1_2][O][C][Branch1_2][C][=O][C][C][C][C][C][C][C][C][C][C][C][C][C][C][C]	[O][=C][Branch2_1][Ring1][=C][O][C][=C][C][=C][C][Branch1_2][N][=C][Ring1][Branch1_2][O][C][Branch1_2][C][=O][C][C][C][C][C][C][C][C][C][C][C][C][C][C][C]:	0.93	O=C(OC1=CC=CC(=C1OC(=O)CCC)CCCCCCCCC)CCC	O=C(OC1=CC=CC(=C1OC(=O)CCC)CCCCCCCCCC)CC	1.0

## Conclusion

With this work, purely data-driven deep learning models for translation between different chemical entity representations are reported. We show that deep learning models are able to capture the essence of SMILES to IUPAC name string conversion (and vice versa) with reaching the 90% accuracy threshold. Despite this promising finding, any large scale and uncurated application should be currently handled with care.

With more data and additional training epochs STOUT is expected to further improve its prediction accuracy in the future. At best, it may finally play in the ballpark of the rule-based systems which further on define the possible top performance. Using the TPU platform will enable the models to be trained in an acceptable amount of time in the order of a few weeks. In addition, STOUT may be extended to alternative sophisticated models used in language translation and understanding, such as BERT [25].

During our revisions, there were two similar preprints, Struct2IUPAC [26] and Translating the Molecules [27], which has been published, reflecting an increase of interest in the translation of SMILES into IUPAC names and vice versa.

## Appendix

BLEU scoring for machine translations is a scoring metric introduced in 2002 used to compare a predicted sentence with the original sentence. Each predicted word is compared with the original, and each word is called an unigram or a 1-gram. In longer sentences we can also compare word pairs or bigrams. Here, we calculated BLEU-1 for unigram comparison, BLEU-2 for the bigram comparison, BLEU-3 for 3-gram comparison and BLEU-4 for 4-gram comparison.

In order to compare the predicted IUPAC name with the original IUPAC name a sentence to sentence comparison should be done, so we used the sentence BLEU scoring function inbuilt in Python Natural Language Toolkit [28]. We use the original IUPAC name as the reference string and the predicted IUPAC name as the candidate string to calculate the BLEU scores.

For all BLEU calculations we used the NLTK sentence BLEU scoring function [24].

Weight distributions for different BLEU scores,

- BLEU-1: weights = (1.0, 0, 0, 0)
- BLEU-2: weights = (0.5, 0.5, 0, 0)
- BLEU-3: weights = (0.3, 0.3, 0.3, 0)
- BLEU-4: weights = (0.25, 0.25, 0.25, 0.25).

BLEU score can reduce according to the following,

- each wrong word match
- each wrong n-gram matches
- length of the candidate string is longer/shorter than reference string
- order of the predicted words are wrong.

For these a penalty will be awarded so the overall score will decrease. A few examples are given below.

Reference: 1,3,7-trimethylpurine-2,6-dione

Candidate: 1,3,7-trimethylpurine-2,6-dione

BLEU score: **1.0**

BLEU-1: 1.00

BLEU-2: 1.00

BLEU-3: 1.00

BLEU-4: 1.00

### Wrong word

Reference: 1,3,7-tri methyl purine-2,6-di one

Candidate: 1,3,7-tri methyl purine-2,6-tri one

BLEU score: **0.87**

BLEU-1: 0.94

BLEU-2: 0.90

BLEU-3: 0.90

BLEU-4: 0.88

### Wrong word pair

Reference: 1,3,7-tri methyl purine-2,6-di one

Candidate: 1,3,7-tri methyl purine-2,6,tri one

BLEU score: **0.81**

BLEU-1: 0.88

BLEU-2: 0.84

BLEU-3: 0.84

BLEU-4: 0.81

### Shorter prediction

Reference: 1,3,7-tri methyl purine-2,6-di one

Candidate: 1,3,7-tri methyl purine-2

BLEU score: **0.63**

BLEU-1: 0.63

BLEU-2: 0.63

BLEU-3: 0.63

BLEU-4: 0.63

### Longer prediction

Reference: 1,3,7-tri methyl purine-2,6-di one

Candidate: 1,3,7-tri methyl purine-2,6-di one, 6-di one, 6-di one

BLEU score: **0.52**

BLEU-1: 0.63

BLEU-2: 0.59

BLEU-3: 0.59

BLEU-4: 0.52

### Wrong order of predictions

Reference: 1,3,7-tri methyl purine-2,6-di one

Candidate: 1,3,7-tri methyl purine-6,2-di one

BLEU score: **0.71**

BLEU-1: 1.00

BLEU-2: 0.86

BLEU-3: 0.80

BLEU-4: 0.71

For the BLEU score calculation, we are using the default settings of sentence BLEU. This corresponds to a four-gram comparison. The weights are distributed evenly. In very few cases as reported in the Results section, we encountered the predictions with BLEU 1.0 where the strings were not identical. The problem can be rectified using more N-gram comparisons with different weight distributions. In our results these cases were very low in number so we used the default settings.

Reference: 4-[(4-amino-2,3,**6**-tri methyl phenyl) methyl]-2,3,**5**-tri methyl aniline

Candidate: 4-[(4-amino-2,3,**5**-tri methyl phenyl)methyl]-2,3,**6**-tri methyl aniline

With sentence BLEU, 4-gram (weights = (0.25,0.25,0.25,0.25))

BLEU score: 1.00

With sentence BLEU, 5-gram (weights = (0.2,0.2,0.2,0.2,0.2))

BLEU score: 0.98

With sentence BLEU, 8-gram (weights = (0.125,0.125,0.125,0.125,0.125,0.125,0.125,0.125))

BLEU score: 0.88.

## Data Availability

The code for STOUT and the trained models are available at https://github.com/Kohulan/Smiles-TO-iUpac-Translator.

## Abbreviations

BLEU: BilinguaL Evaluation Understudy
BERT: Bidirectional encoder representations from transformers
CDK: Chemistry development kit
DECIMER: Deep lEarning for Chemical Image Recognition
FTP: File transfer protocol
GPU: Graphics processing unit
IUPAC: International Union of Pure and Applied Chemistry
InChI: International chemical identifier
NMT: Neural machine translation
OPSIN: Open parser for systematic IUPAC nomenclature
RAM: Random access memory
RNN: Recurrent neural network
SDF: Structure data file
SELFIES: Self-referencing embedded strings
SMARTS: SMILES arbitrary target specification
SMILES: Simplified molecular-input line-entry system
STOUT: Smiles TO iUpac Translator
TPU: Tensor processing units
TFRecord: TensorFlow Record file
VRAM: Video random access memory
